# Obesity is associated with pain and impaired mobility despite therapy in systemic lupus erythematosus

**DOI:** 10.3389/fmed.2023.1247354

**Published:** 2023-08-24

**Authors:** Alexander Borg, Julius Lindblom, Alvaro Gomez, Ameneh Soltani, Yvonne Enman, Emelie Heintz, Malin Regardt, David Grannas, Sharzad Emamikia, Ioannis Parodis

**Affiliations:** ^1^Division of Rheumatology, Department of Medicine Solna, Karolinska Institutet and Karolinska University Hospital, Stockholm, Sweden; ^2^Department of Learning, Informatics, Management and Ethics (LIME), Karolinska Institutet, Stockholm, Sweden; ^3^Department of Neurobiology, Care Sciences and Society, Karolinska Institutet, Stockholm, Sweden; ^4^Occupational Therapy and Physiotherapy, Karolinska University Hospital, Stockholm, Sweden; ^5^Division of Biostatistics, Institute of Environmental Medicine, Karolinska Institutet, Stockholm, Sweden; ^6^Department of Rheumatology, Faculty of Medicine and Health, Örebro University, Örebro, Sweden

**Keywords:** systemic lupus erythematosus, body mass index, health-related quality of life, patient reported outcomes, patient perspective

## Abstract

**Objective:**

To investigate whether abnormal BMI is associated with health-related quality of life (HRQoL) impairments, defined as patient-reported problems within the different dimensions of the three-level EQ-5D (EQ-5D-3L), before and after treatment for active systemic lupus erythematosus (SLE).

**Patients and methods:**

We conducted a post-hoc analysis of data from two phase III clinical trials of belimumab in SLE, i.e., BLISS-52 (*n* = 865) and BLISS-76 (*n* = 819). Underweight was defined as BMI <18.5 kg/m^2^, normal weight as BMI ≥18.5 but <25 kg/m^2^, pre-obesity as BMI ≥25 but <30 kg/m^2^, and obesity as BMI ≥30 kg/m^2^. We investigated associations between BMI groups and problems (level 2 or 3) within each one of the five EQ-5D dimensions before treatment initiation and at week 52, using logistic regression analysis adjusting for age, ethnicity, disease activity, and glucocorticoid dose, and for the post-treatment analysis also for belimumab treatment and baseline EQ-5D-3L responses.

**Results:**

Of 1,684 patients included, 73 (4%) were classified as underweight, 850 (50%) as normal weight, 438 (26%) as pre-obese, and 323 (19%) as obese. At baseline, obesity was associated with mild to severe problems in all EQ-5D dimensions (*p* < 0.05 for all), yielding the strongest association with problems in mobility (adjusted odds ratio, aOR: 2.1; 95% confidence interval, CI: 1.6–2.8; *p* < 0.001). Pre-obesity was also associated with problems in mobility (aOR: 1.4; 95% CI: 1.1–1.8; *p* = 0.005). Post-intervention, obesity was associated with problems in mobility and pain/discomfort, and pre-obesity with problems in mobility and self-care (*p* < 0.05 for all).

**Conclusion:**

Our study adds to the evidence that high BMI negatively affects SLE patients’ HRQoL, with obesity being associated with pain and impaired mobility despite therapy.

## 1. Introduction

Systemic lupus erythematosus (SLE) is a chronic autoimmune disease that commonly affects the skin, joints, kidneys, and central nervous system (CNS), being characterized by an immense heterogeneity in terms of clinical presentation (1). SLE causes severe impairments of health-related quality of life (HRQoL) (1), a multi-domain and multifactorial concept (2) that relates to the impact of a disease and its medications on individuals’ perceived well-being in physical, mental, and social aspects of life (3). The importance of assessing HRQoL in clinical trials in the form of patient-reported outcome (PRO) measures was highlighted during the Outcome Measures in Rheumatology (OMERACT) IV consensus conference in 1998 (4) and is now requested by the American Food and Drug Administration (FDA) (5).

Survival has improved considerably for people with SLE over the past decades, but the patients still experience a poorer HRQoL compared with the general population (6). Comparing the impact of SLE with other chronic diseases such as type 2 diabetes and hypertension, SLE patients generally experience lower HRQoL both in physical and mental aspects (6), with fatigue being one of the most common complaints (7). Diminutions in different aspects of HRQoL have been shown to be attributable to the daily glucocorticoid dose, depression, fatigue, and high body mass index (BMI), independently of the effect of SLE disease activity or organ damage (8, 9).

Obesity is a factor that contributes to HRQoL impairments (9–11), and is associated with poor functional capacity and increased inflammation in SLE patients (12). In previous studies (13, 14), we investigated the association between BMI and HRQoL using the Medical Outcomes Study (MOS) 36-item Short Form (SF-36) (15), Functional Assessment of Chronic Illness Therapy Fatigue (FACIT-F) (16), and the index score and visual analogue scale of the three-level EQ-5D (EQ-5D-3L). We showed that SLE patients with BMI above normal experienced a lower HRQoL regarding physical aspects, fatigue, and social functioning compared with patients of normal weight, independently of demographic and disease-related factors (13). Furthermore, pre-obesity and obesity were associated with adverse physical and mental HRQoL outcomes following therapy for active SLE, while underweight was associated with adverse mental HRQoL outcomes (14). However, the sensitivity of the five different dimensions of the EQ-5D to detect differences across BMI categories before and after therapeutic intervention for active SLE has not been investigated. Such observations are informative for optimizing the use of the different HRQoL instruments in clinical trials and observational studies.

The aim of the present study was to investigate associations between SLE patients’ BMI and their experiences of HRQoL based on responses to the different dimensions of EQ-5D-3L before and after therapeutic intervention for active SLE.

## 2. Materials and methods

### 2.1. Study population

We analyzed data collected in the setting of two phase III randomized controlled trials that evaluated the efficacy of belimumab in patients with SLE, i.e., the BLISS- 52 (17) and BLISS-76 (18) clinical trials. BLISS-52 comprised 865 patients with SLE from Eastern Europe, Asia and Latin America, and BLISS-76 comprised 819 SLE patients from Europe and North/Central America.

The trials had a similar design and identical efficacy endpoints, i.e., attainment of Systemic Lupus Erythematosus Responder Index 4 (SRI-4) response at week 52. Inclusion criteria comprised SLE diagnosis in accordance with the revised American College of Rheumatology (ACR) criteria (19), age ≥ 18 years, score of ≥6 in the Safety of Estrogens in Lupus Erythematosus National Assessment – Systemic Lupus erythematosus Disease Activity Index (SELENA-SLEDAI) (20) at screening, positive anti-nuclear antibody (ANA) titres (≥1:80) and/or anti-double stranded DNA (anti-dsDNA) antibody levels (≥30 IU/mL). All participating patients were required to be on stable non-biological standard therapy (ST) for at least 30 days preceding initiation of the trial, comprising glucocorticoids, antimalarial agents (AMA), non-steroidal anti-inflammatory drugs, and/or immunosuppressive agents at fixed tolerable doses. Pregnant patients were excluded, as were those with severe active lupus nephritis or central nervous system (CNS) lupus, those who had received intravenous cyclophosphamide during the last 6 months preceding the trial initiation or intravenous immunoglobulin or prednisone equivalents at doses >100 mg/day during the last 3 months, and finally patients with any experience of previous B cell targeting therapy.

While the BLISS-52 and BLISS-76 trials evaluated the efficacy of belimumab versus placebo on top of non-biological ST, we analyzed the potential impact of BMI as an independent factor on SLE patients’ HRQoL at active disease, i.e., prior to commencement of therapeutic intervention, as well as 52 weeks after treatment commencement. Data from BLISS-52 and BLISS-76 were made available by GlaxoSmithKline (GSK; Uxbridge, UK) through the Clinical Study Data Request (CSDR) portal.

### 2.2. Body mass index classification

BMI was the main exposure of the current analysis. For analyses before the trial intervention, we used BMI data at baseline, whereas for analyses after the trial intervention, we used average BMI throughout the trial. The average BMI was calculated by dividing the sum of the BMI scores from all study visits by the number of study visits. A total of 15 study visits at regular intervals were available from study initiation through week 52. Patients were classified based on their BMI according to the World Health Organization (WHO) classification system (21); underweight was defined as BMI <18.5 kg/m^2^, normal weight as BMI ≥18.5 but <25 kg/m^2^, pre-obesity as BMI ≥25 but <30 kg/m^2^, and obesity as BMI ≥30 kg/m^2^.

### 2.3. Assessment of HRQoL by EQ-5D

The EQ-5D health questionnaire consists of two parts, a descriptive system and a visual analogue scale (VAS). The descriptive system assesses health within five dimensions, i.e., mobility (MOB), self-care (SC), usual activities (UA), pain/discomfort (PD), and anxiety/depression (AD). In the EQ-5D-3L version, each of these dimensions has three levels, indicating no problems (level 1), some/moderate problems (level 2), and severe/extreme problems (level 3). To determine a unique overall health status, reported levels from each one of the five dimensions are combined and converted into a summary index score. In the present study, HRQoL impairments were defined as experiencing some/moderate or severe/extreme problems (i.e., level 2 or level 3) in each one of the five dimensions of the descriptive system of EQ-5D-3L. We explored associations between abnormal BMI and HRQoL impairments at baseline and at week 52.

### 2.4. Statistics

Data are presented as numbers (percentage) or the mean (standard deviation). Comparisons were performed between patients reporting no problems and patients reporting HRQoL impairments, using logistic regression analysis. Patients of normal weight formed the reference BMI group. Results are reported as the odds ratio (OR), 95% confidence interval (CI), and *p-*value for each comparison. Baseline associations derived from univariable logistic regression models. Associations at week 52 derived from multivariable logistic regression models accounting for baseline EQ-5D-3L responses. We termed results from the above models “unadjusted.”

Next, we employed multivariable logistic regression models to account for potential confounding factors; covariates in the models were selected *a priori* and included age, ethnicity, SLE disease activity measured using the SLE Disease Activity Index 2000 (SLEDAI-2 K) (22), and prednisone (or equivalent) dose. To investigate the independent effect of BMI on EQ-5D dimensions irrespective of treatment and patients’ baseline EQ-5D status, we also adjusted results at week 52 for belimumab treatment and baseline EQ-5D-3L responses. We termed results from the above models “adjusted.”

Missing BMI values were imputed using the BMI of the previous and next available visits, and for missing values from the last visits we applied the last observation carried forward (LOCF) method. Of the total BLISS population (*N* = 1,684), 1665 patients had available EQ-5D-3L data at week 52 and hence comprised the post-treatment population of the present study. The number of patients with available EQ-5D-3L data at baseline differed across the different EQ-5D dimensions, comprising 1655 patients with available data for the anxiety/depression dimension, 1652 for the mobility and pain/discomfort dimensions, 1651 for the self-care dimension, and 1650 for the usual activities dimension.

**Figure 1:**
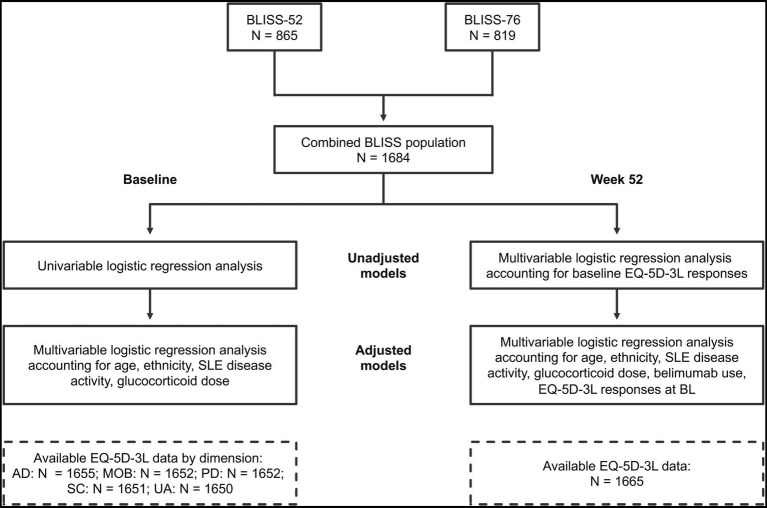
Study workflow. Schematic illustration of the study workflow and study population. BL, baseline; BMI, body mass index; MOB, mobility; SC, self-care; UA, usual activities; PD, pain/discomfort; AD, anxiety/depression.

Results yielding *p* values <0.05 were considered statistically significant. The R Statistics software version 4.1.0 (R Foundation for Statistical Computing, Vienna, Austria) was used for statistical analyses. The analysis workflow and study population are depicted in Figure 1.

### 2.5. Ethics statement

Written informed consent was obtained from all participants prior to enrolment in the trials (17, 18). Ethical permissions in accordance with the principles of the declaration of Helsinki were acquired at all participating sites, and our study questions were assessed and approved by a committee within GSK before access to data was granted. The study protocol for this post-hoc analysis was reviewed and approved by the Swedish Ethical Review Authority (2019–05498).

## 3. Results

### 3.1. Patient characteristics

Characteristics of the combined BLISS population (*N* = 1,684) are summarized in Table 1. At baseline, 873 (51.8%) patients had a normal BMI, while 80 (4.8%) patients were underweighted, 435 (25.8%) patients were pre-obese, and 296 (17.6%) were obese. At week 52, 850 (50.5%) patients were normal-weighted, 73 (4.3%) were underweighted, 438 (26%) were pre-obese, and 323 (19.2%) were obese. The different BMI groups had similar SLEDAI-2 K scores before and after the trial intervention, received similar prednisone equivalent doses at baseline, and were allocated to the different study arms in a balanced manner. However, mean age was higher in groups with higher BMI, and obese patients were more frequently of Caucasian ancestry and less frequently of Asian ancestry than groups with lower BMI.

**Table 1:** Patient characteristics.

	Underweight (*N* = 73)	Normal weight (*N* = 850)	Pre-obesity (*N* = 438)	Obesity (*N* = 323)	Total (*N* = 1,684)
Demographics					
Age (years)	31.0 ± 9.8	35.3 ± 10.9	39.7 ± 11.3	43.4 ± 11.0	37.8 ± 11.5
Female sex	70 (95.9%)	808 (95.1%)	404 (92.2%)	303 (93.8%)	1585 (94.1%)
Ancestry
Asian	32 (43.8%)	237 (27.9%)	71 (16.2%)	15 (4.6%)	355 (21.1%)
Black/African American	6 (8.2%)	43 (5.1%)	49 (11.2%)	50 (15.5%)	148 (8.8%)
Indigenous American*	14 (19.2%)	193 (22.7%)	115 (26.3%)	61 (18.9%)	383 (22.7%)
White/Caucasian	21 (28.7%)	377 (44.4%)	203 (46.3%)	197 (61.0%)	798 (47.4%)
Clinical data					
Mean BMI (week 0–52)	17.7 ± 0.8	22.0 ± 1.8	27.2 ± 1.4	35.4 ± 4.9	25.8 ± 5.9
SLEDAI-2 K score
Baseline	10.5 ± 4.1	9.8 ± 3.8	10.1 ± 3.7	10.1 ± 4.0	10.0 ± 3.8
Week 52	6.8 ± 5.1	6.1 ± 4.1	6.0 ± 4.5	6.2 ± 4.7	6.2 ± 4.4
SDI score
Baseline	0.7 ± 1.1	0.6 ± 1.1	0.8 ± 1.3	1.1 ± 1.5	0.8 ± 1.2
Week 52	0.8 ± 1.1	0.7 ± 1.1	0.9 ± 1.3	1.2 ± 1.5	0.8 ± 1.3
Trial treatment:
Placebo	21 (28.8%)	296 (34.8%)	142 (32.4%)	103 (31.9%)	562 (33.4%)
Belimumab 1 mg/kg	29 (39.7%)	269 (31.6%)	154 (35.2%)	107 (33.1%)	559 (33.2%)
Belimumab 10 mg/kg	23 (31.5%)	285 (33.5%)	142 (32.4%)	113 (35.0%)	563 (33.4%)
Prednisone use	69 (94.5%)	755 (88.8%)	380 (86.8%)	249 (77.1%)	1453 (86.3%)
Prednisone eq. dose at baseline (mg/day)	10.6 ± 7.3	11.0 ± 8.5	11.1 ± 9.2	9.9 ± 8.6	10.8 ± 8.7
IS use	33 (45.2%)	392 (46.1%)	220 (50.2%)	175 (54.2%)	820 (48.7%)
Azathioprine	16 (21.9%)	195 (22.9%)	105 (24.0%)	73 (22.6%)	389 (23.1%)
Methotrexate	8 (11.0%)	112 (13.2%)	52 (11.9%)	59 (18.3%)	231 (13.7%)
Mycophenolic acid	8 (11.0%)	83 (9.8%)	56 (12.8%)	42 (13.0%)	189 (11.2%)
Other IS^‡^	1 (1.4%)	17 (2.0%)	9 (2.1%)	6 (1.9%)	33 (2.0%)
Antimalarial agent use^†^	54 (74.0%)	576 (67.8%)	269 (61.4%)	201 (62.2%)	1100 (65.3%)

Data are presented as numbers (percentage) or the mean ± standard deviation. BMI, body mass index; eq., equivalent; IS, immunosuppressant; SDI, Systemic Lupus International Collaborating Clinics (SLICC)/American College of Rheumatology (ACR) Damage Index; SLE, systemic lupus erythematosus; SLEDAI-2 K: SLE Disease Activity Index 2000. *Alaska Native or American Indian from North, South or Central America. ^†^Hydroxychloroquine, chloroquine, mepacrine, mepacrine hydrochloride, or quinine sulfate. ^‡^Cyclosporine, oral cyclophosphamide, leflunomide, mizoribine, or thalidomide.

### 3.2. HRQoL impairments between BMI groups at baseline

At baseline, proportions of patients reporting problems did not differ between underweighted and normal-weight patients within any of the EQ-5D dimensions (Table 2). Proportions of patients reporting HRQoL impairments were greater among pre-obese compared with normal-weight patients regarding MOB, SC, UA, and PD, but not AD, with the highest difference found for MOB (47.1% versus 35.4%; OR: 1.63; 95% CI: 1.28–2.06; *p* < 0.001) and PD (83.2% versus 75.2%; OR: 1.63; 95% CI: 1.21–2.19; *p* = 0.001; Table 2). Proportions of patients reporting problems were greater among obese compared with normal-weight patients within all EQ-5D dimensions; the differences between obese and normal-weight patients were more pronounced than those between pre-obese and normal-weight patients (Table 2).

**Table 2:** Associations between abnormal BMI and HRQoL impairments at baseline.

	BMI categories			
	Normal weight (*N* = 854)	Underweight (*N* = 76)	Pre-obese (*N* = 427)	Obese (*N* = 295)
EQ-5D mobility				
Level 2 + Level 3	297 + 5 = 302 (35.4%)	25 + 1 = 26 (34.2%)	200 + 1 = 201 (47.1%)	173 + 1 = 174 (58.9%)
Unadj. OR (95% CI)	Ref.	0.95 (0.58–1.56); *p* = 0.840	**1.63 (1.28–2.06);** ***p* < 0.001**	**2.63 (2.00–3.45);** ***p* < 0.001**
Adj. OR (95% CI)	Ref.	1.04 (0.62–1.71); *p* = 0.874	**1.42 (1.11–1.82);** ***p* = 0.005**	**2.09 (1.57–2.79);** ***p* < 0.001**
EQ-5D self-care				
Level 2 + Level 3	123 + 5 = 128 (15.0%)	16 + 1 = 17 (22.1%; *N* = 77)	87 + 1 = 88 (20.7%; *N* = 426)	72 + 5 = 77 (26.2%; *N* = 294)
Unadj. OR (95% CI)	Ref.	1.61 (0.91–2.84); *p* = 0.100	**1.48 (1.09–1.99);** ***p* = 0.011**	**2.01 (1.46–2.77);** ***p* < 0.001**
Adj. OR (95% CI)	Ref.	1.71 (0.93–3.01); *p* = 0.074	1.30 (0.95–1.77); *p* = 0.099	**1.63 (1.15–2.28):** ***p* = 0.005**
EQ-5D usual activities				
Level 2 + Level 3	404 + 22 = 426 (50.0%; *N* = 852)	36 + 2 = 38 (49.4%; *N* = 77)	237 + 16 = 253 (59.4%; *N* = 426)	197 + 18 = 215 (72.9%)
Unadj. OR (95% CI)	Ref.	0.97 (0.61–1.55); *p* = 0.913	**1.46 (1.16–1.85);** ***p* = 0.002**	**2.69 (2.01–3.59);** ***p* < 0.001**
Adj. OR (95% CI)	Ref.	1.06 (0.65–1.73); *p* = 0.811	1.26 (0.98–1.61); *p* = 0.070	**2.04 (1.50–2.78);** ***p* < 0.001**
EQ-5D pain/discomfort				
Level 2 + Level 3	580 + 60 = 640 (75.2%; *N* = 851)	58 + 3 = 61 (79.2%; *N* = 77)	306 + 50 = 356 (83.2%; *N* = 428)	220 + 46 = 266 (89.9%; *N* = 296)
Unadj. OR (95% CI)	Ref.	1.26 (0.71–2.23); *p* = 0.433	**1.63 (1.21–2.19);** ***p* = 0.001**	**2.92 (1.94–4.40);** ***p* < 0.001**
Adj. OR (95% CI)	Ref.	1.50 (0.85–2.78); *p* = 0.181	1.30 (0.96–1.78); *p* = 0.099	**1.87 (1.23–2.91);** ***p* = 0.004**
EQ-5D anxiety/depression				
Level 2 + Level 3	404 + 47 = 451 (52.7%; *N* = 855)	33 + 4 = 37 (48.1%; *N* = 77)	209 + 27 = 236 (55.1%; *N* = 428)	162 + 17 = 179 (60.7%)
Unadj. OR (95% CI)	Ref.	0.83 (0.60–1.32); *p* = 0.429	1.10 (0.87–1.39); *p* = 0.418	**1.38 (1.06–1.81);** ***p* = 0.018**
Adj. OR (95% CI)	Ref.	0.82 (0.51–1.31); *p* = 0.404	1.09 (0.86–1.39); *p* = 0.485	**1.34 (1.01–1.79);** ***p* = 0.043**

Data are presented as numbers (percentage). In case of missing data, the number of available observations is indicated with *N*. *P*-values were derived from logistic regression analysis. *P*-values < 0.05 are in bold. Adj., adjusted; BMI, body mass index; CI, confidence interval; HRQoL, health-related quality of life; OR, odds ratio; Ref, reference; Unadj., unadjusted.

**Figure 2:**
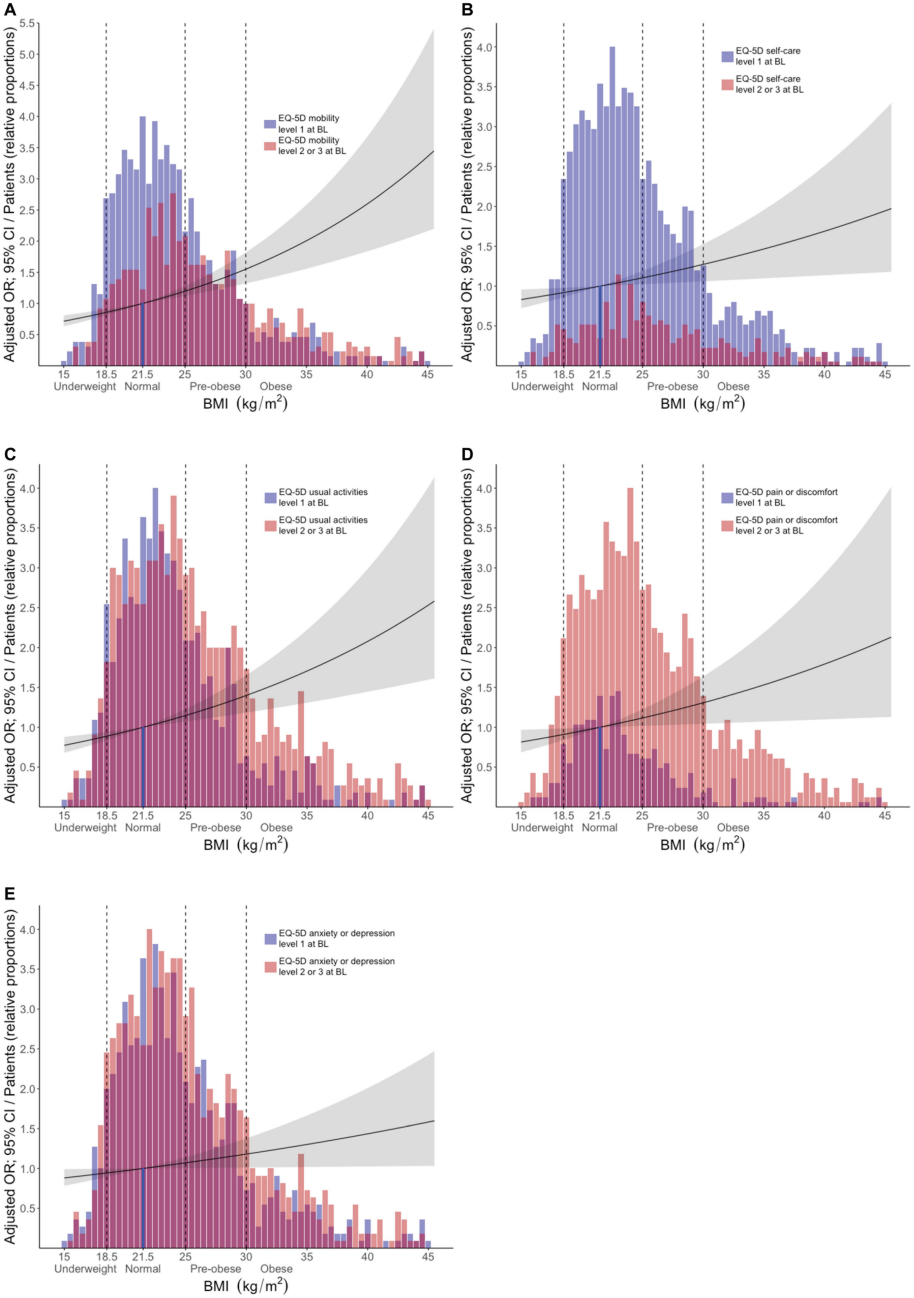
Associations between BMI and problems across different EQ-5D dimensions at baseline. The bars delineate relative proportions of patients who reported mild/moderate or severe/extreme problems in light red color, or no problems in blue color, within the **(A)** mobility, **(B)** self-care, **(C)** usual activities, **(D)** pain/discomfort, and **(E)** anxiety/depression, across BMI categories. Purple color indicates overlap between the two categories (light red and blue). The reference line indicates the average normal BMI (21.5 kg/m^2^). Black lines represent exponentiated odds ratios and grey areas indicate the corresponding 95% confidence interval for each instance, with the average normal BMI as the reference comparator. These metrics were generated from multivariable logistic regression analysis, in models where apart from the different BMI categories, age, ancestry, SLE disease activity, and average glucocorticoid dose were also included as covariates. The *x* axis denotes BMI (kg/m^2^) and the *y* axis denotes the adjusted OR (exponentiated). The relative proportion of patients for each bar was calculated based on the following formula: number of patients within the respective BMI range/(maximum number of patients within any BMI range × 0.25). BL, baseline; BMI, body mass index; MOB, mobility; SC, self-care; UA, usual activities; PD, pain/discomfort; AD, anxiety/depression.

In adjusted models, increasing BMI (using BMI as a continuous variable) was associated with experience of problems in all EQ-5D-3L dimensions; results are detailed in Supplementary Table S1. In comparisons between abnormal BMI categories and normal-weight patients, pre-obesity was associated with experience of problems regarding MOB (OR: 1.42; 95% CI: 1.11–1.82; *p* = 0.005), while obesity was associated with experience of problems in all EQ-5D dimensions, i.e., MOB (OR: 2.09; 95% CI: 1.57–2.79; *p* < 0.001), SC (OR: 1.63; 95% CI: 1.15–2.28; p = 0.005), UA (OR: 2.04; 95% CI: 1.50–2.78; *p* < 0.001), PD (OR: 1.87; 95% CI: 1.23–2.91; *p* = 0.004), and AD (OR: 1.34; 95% CI: 1.01–1.79; *p* = 0.043). We observed no associations between underweight and HRQoL impairments. Results are detailed in Supplementary Table S2 and illustrated in Figure 2.

### 3.3. HRQoL impairments between BMI groups post-treatment

Associations between abnormal BMI and experience of problems post-treatment accounted for baseline EQ-5D-3L responses, as described above. Proportions of patients reporting problems did not differ between underweighted and normal-weight patients in any of the EQ-5D dimensions (Table 3). Proportions of patients reporting problems at week 52 were greater in pre-obese compared with normal-weight patients with regard to MOB (38.9% versus 26.6%; OR: 1.63; 95% CI: 1.24–2.15; *p* < 0.001), SC (19.7% versus 12.5%; OR: 1.63; 95% CI: 1.14–2.33; *p* = 0.007), and UA (47.9% versus 38.4%; OR: 1.39; 95% CI: 1.07–1.80; *p* = 0.013), but not PD or AD (Table 3). Lastly, proportions of patients reporting problems were greater among obese compared with normal-weight patients regarding MOB, SC, UA, and PD, but not AD, with the greatest differences being observed within MOB (47.1% versus 26.6%; OR: 1.82; 95% CI: 1.35–2.45; *p* < 0.001) and PD (82.4% versus 64.3%; OR: 2.07; 95% CI: 1.49–2.92; *p* < 0.001; Table 3).

**Table 3:** Associations between abnormal BMI and HRQoL impairments at week 52.

	BMI categories			
	Normal weight (*N* = 838)	Underweight (*N* = 72)	Pre-obese (*N* = 432)	Obese (*N* = 323)
EQ-5D mobility				
Level 2 + Level 3	218 + 5 = 223 (26.6%)	13 + 2 = 15 (20.8%)	163 + 5 = 168 (38.9%)	152 + 0 = 152 (47.1%)
Unadj. OR (95% CI)	Ref.	0.75 (0.39–1.40); *p* = 0.382	**1.63 (1.24–2.15);** ***p* < 0.001**	**1.82 (1.35–2.45);** ***p* < 0.001**
Adj. OR (95% CI)	Ref.	0.92 (0.46–1.73); *p* = 0.796	**1.47 (1.10–1.96);** ***p* = 0.009**	**1.42 (1.03–1.96);** ***p* = 0.031**
EQ-5D self-care				
Level 2 + Level 3	100 + 5 = 105 (12.5%)	10 + 2 = 12 (16.7%)	82 + 3 = 85 (19.7%)	69 + 3 = 72 (22.3%)
Unadj. OR (95% CI)	Ref.	1.46 (0.67–2.97); *p* = 0.317	**1.63 (1.14–2.33);** ***p* = 0.007**	**1.55 (1.05–2.27);** ***p* = 0.026**
Adj. OR (95% CI)	Ref.	1.67 (0.75–3.47); *p* = 0.187	**1.52 (1.05–2.19);** ***p* = 0.027**	1.27 (0.84–1.91); *p* = 0.236
EQ-5D usual activities				
Level 2 + Level 3	306 + 16 = 322 (38.4%)	24 + 2 = 26 (36.1%)	191 + 16 = 207 (47.9%)	175 + 10 = 185 (57.3%)
Unadj. OR (95% CI)	Ref.	1.03 (0.59–1.78); *p* = 0.913	**1.39 (1.07–1.80);** ***p* = 0.013**	**1.61 (1.21–2.14);** ***p* = 0.001**
Adj. OR (95% CI)	Ref.	1.23 (0.70–2.15); *p* = 0.468	1.22 (0.93–1.60); *p* = 0.158	1.18 (0.87–1.61); *p* = 0.284
EQ-5D pain/discomfort				
Level 2 + Level 3	493 + 46 = 539 (64.3%)	40 + 4 = 44 (61.1%)	273 + 29 = 302 (69.9%)	232 + 34 = 266 (82.4%)
Unadj. OR (95% CI)	Ref.	0.80 (0.47–1.37); *p* = 0.399	1.12 (0.860–1.47); *p* = 0.396	**2.07 (1.49–2.92);** ***p* < 0.001**
Adj. OR (95% CI)	Ref.	0.92 (0.53–1.59); *p* = 0.749	0.99 (0.75–1.30); *p* = 0.914	**1.53 (1.08–2.20);** ***p* = 0.018**
EQ-5D anxiety/depression				
Level 2 + Level 3	332 + 40 = 372 (44.4%)	30 + 4 = 34 (47.2%)	189 + 17 = 206 (47.7%)	144 + 25 = 169 (52.3%)
Unadj. OR (95% CI)	Ref.	1.34 (0.77–2.32); *p* = 0.296	1.11 (0.86–1.45); *p* = 0.426	1.29 (0.97–1.73); *p* = 0.084
Adj. OR (95% CI)	Ref.	1.45 (0.83–2.54); *p* = 0.189	1.04 (0.79–1.37); *p* = 0.767	1.12 (0.82–1.53); *p* = 0.471

Data are presented as numbers (percentage). In case of missing data, the number of available observations is indicated with *N*. *P*-values were derived from logistic regression analysis. *P*-values < 0.05 are in bold. Adj., adjusted; BMI, body mass index; CI, confidence interval; HRQoL, health-related quality of life; OR, odds ratio; Ref, reference; Unadj., unadjusted.

**Figure 3:**
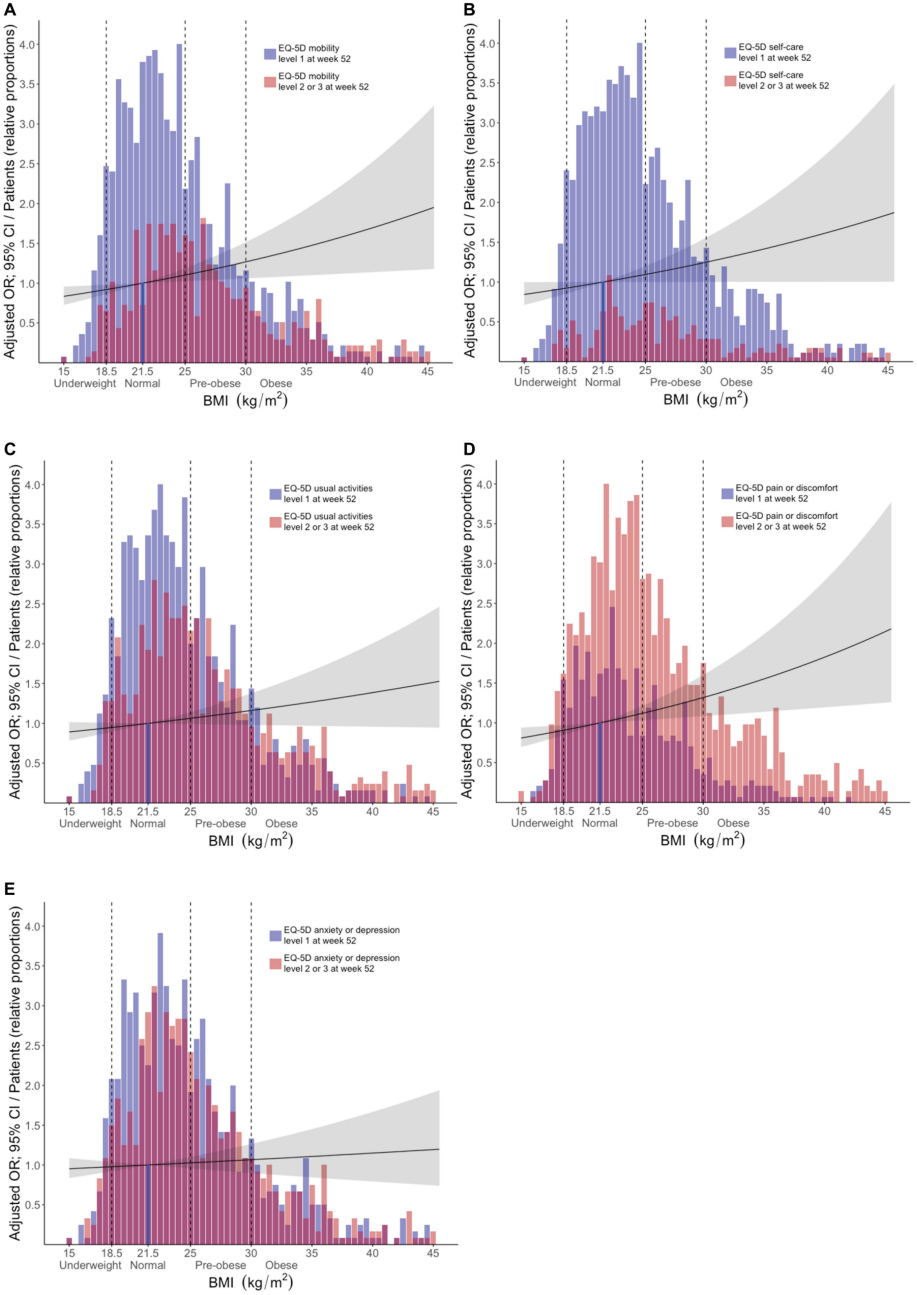
Associations between BMI and problems across different EQ-5D dimensions post-treatment. The bars delineate relative proportions of patients who reported mild/moderate or severe/extreme problems in light red color, or no problems in blue color, within the **(A)** mobility, **(B)** self-care, **(C)** usual activities, **(D)** pain/discomfort, and **(E)** anxiety/depression, across BMI categories. Purple color indicates overlap between the two categories (light red and blue). The reference line indicates the average normal BMI (21.5 kg/m^2^). Black lines represent exponentiated odds ratios and grey areas indicate the corresponding 95% confidence interval for each instance, with the average normal BMI as the reference comparator. These metrics were generated from multivariable logistic regression analysis, in models where apart from the different BMI categories, age, ancestry, SLE disease activity, average glucocorticoid dose, baseline EQ-5D responses, and belimumab use were also included as covariates. The *x* axis denotes BMI (kg/m^2^) and the *y* axis denotes the adjusted OR (exponentiated). The relative proportion of patients for each bar was calculated based on the following formula: number of patients within the respective BMI range/(maximum number of patients within any BMI range × 0.25). BMI, body mass index; MOB, mobility; SC, self-care; UA, usual activities; PD, pain/discomfort; AD, anxiety/depression.

In adjusted models, increasing BMI (using BMI as a continuous variable) was associated with experience of problems regarding MOB (OR: 1.03; 95% CI: 1.01–1.05; *p* = 0.009), SC (OR: 1.03; 95% CI: 1.00–1.05; *p* = 0.048), and PD (OR: 1.03; 95% CI: 1.01–1.06; *p* = 0.005), but not regarding UA or AD. Results are detailed in Supplementary Table S3. In stratified analysis, pre-obesity was associated with experience of problems within the MOB (OR: 1.47; 95% CI: 1.10–1.96; p = 0.009) and SC (OR: 1.52; 95% CI: 1.05–2.19; *p* = 0.027) dimensions, while obesity was associated with HRQoL impairments within the MOB (OR: 1.42; 95% CI: 1.03–1.96; *p* = 0.031) and PD (OR: 1.53; 95% CI: 1.08–2.20; *p* = 0.018) dimensions. We observed no associations between underweight and report of problems in any EQ-5D dimension. Results are detailed in Supplementary Table S4 and illustrated in Figure 3.

Among covariates other than BMI, high SLEDAI-2 K score was associated with experience of problems regarding all EQ-5D dimensions, both before and after therapy, as was increasing age, the latter however not regarding SC and AD at baseline. Moreover, patients of Asian and Indigenous American ancestry reported problems less frequently compared with White/Caucasian patients within several EQ-5D dimensions, whereas patients of African American origin more frequently reported problems regarding PD compared with White/Caucasian patients (OR: 2.08; 95% CI: 1.13–4.21; *p* = 0.028). Results are detailed in Supplementary Tables S1, S3.

## 4. Discussion

In the present study, we investigated associations between BMI and different aspects of SLE patients’ HRQoL experience assessed using the EQ-5D, both at the time of active disease and after a 52-week long therapeutic intervention. Overall, we found that obese patients with SLE reported problems at a greater extent than normal-weight SLE patients in all EQ-5D dimensions at baseline, independently of demographic and disease-related factors. Importantly, these associations remained significant for the mobility and pain/discomfort dimensions after therapeutic intervention. While no causal relationship between obesity and diminished HRQoL could be concluded from the present study, the results imply that benefits from weight control strategies might be expected, and that such strategies could be considered as an adjunct to appropriate pharmacological therapy in overweight SLE patients.

The observed associations between elevated BMI and impairments in physical aspects of HRQoL, particularly within mobility, are in line with several previous reports (9–12). Similar associations in the same direction have been consistently demonstrated across studies of SLE populations of different age (23), ethnic origin (24), with different degrees of disease activity, from different settings, and with HRQoL being evaluated using different instruments or conceptually different HRQoL outcome measures. Furthermore, we observed gradually stronger relationships with HRQoL impairments with increasing BMI, with obese individuals reporting HRQoL impairments to a greater extent than pre-obese patients, as also shown in our previous reports using other HRQoL instruments, i.e., the SF-36 and FACIT-F (13, 14).

When evaluating the effect of the 52-week therapeutic intervention by comparing measures before and after, we observed reductions in disease activity of similar magnitude across BMI categories, and lower proportions of patients reporting HRQoL impairments. Even though the differences in HRQoL impairments between obese and normal-weight individuals attenuated after the trial intervention, obese patients experienced problems more frequently than normal-weight patients within most EQ-5D dimensions. This highlights the imperative need of accounting for the patient-reported experience in clinical practice, in order to detect patients’ needs that could be overlooked by routine clinical and laboratory measures.

Inflammation has been proposed to be a link between obesity and HRQoL impairments in different disease populations. Obesity has been shown to contribute to a pro-inflammatory state, where activated mononuclear cells produce pro-inflammatory cytokines (25), while excess adipose tissue is believed to induce production of inflammatory cytokines and increased levels of C-reactive protein (CRP) (26). Interestingly, we did not observe significant differences in SLEDAI-2 K scores across BMI categories, either at active disease or after the therapeutic intervention. Nevertheless, the effect of low-grade inflammation driven by the adipose tissue may not be captured by disease activity measures, or at least the ones currently used in SLE, but it could still impact patient perceptions of fatigue, pain, and HRQoL (27).

This study adds to the current body of knowledge an understanding on the ability of the EQ-5D dimensions to detect differences across BMI categories. Importantly, the EQ-5D is a brief instrument, with a low time burden for patients, that has shown satisfactory psychometric properties in the SLE population (28, 29). However, it must be acknowledged that for the calculation of EQ-5D index scores, several different value sets exist for different countries. Thus, when conducting multinational studies that collect EQ-5D data, it is implausible to use the value set that corresponds to each individual patient, and researchers must instead decide on a single value set from a country that is expected to represent individuals from different backgrounds, such as the ones from the United States or the United Kingdom (30, 31). To alleviate this issue, our study made use of the individual EQ-5D dimension scores, which provide more granular and interpretable information of the patients’ HRQoL experience. This may represent a more comprehensive way of utilizing and reporting from the EQ-5D instrument, as a complement to the EQ-5D utility index and VAS scores.

Our study has several limitations. Firstly, no conclusions can be drawn on causality. Moreover, the lack of a comparator group prevented us from addressing whether the observed associations are disease-specific, or rather attributable to obesity in general. Furthermore, we lacked data on comorbidities and patients’ regular diet or potential dietary changes during the study follow-up, which would have allowed us to better account for possible confounding factors and explore possible mechanisms through which obesity may be associated with impaired HRQoL. Moreover, medications and their potential adverse effects may influence weight, and the study participants were on various treatments during the study follow-up. To account for weight changes during the study period, we calculated the mean BMI of the 52-week study period, which was used in all post-treatment analyses, as was use of belimumab. The inclusion criteria of the trials limit the external validity, as active musculoskeletal and mucocutaneous SLE is overrepresented in the study population while severe active renal and neuropsychiatric disease was excluded. Strengths of our study include the large study population and data completeness, which allowed us to adjust for several confounders, the ethnic diversity of the participants, and the possibility to evaluate associations before and after a trial intervention.

In conclusion, we demonstrated that obese patients with SLE experienced mobility problems to a greater extent than normal-weight SLE patients, irrespective of disease activity, both before and after therapeutic intervention. Although corroboration through prospective investigation of the effects of weight-control strategies on SLE patients’ HRQoL is needed, the results of the present study lend support for expected benefits from such strategies along with the appropriate pharmacological therapy in overweight SLE patients.

## Data availability statement

The original contributions presented in the study are included in the article/Supplementary material, further inquiries can be directed to the corresponding author.

## Ethics statement

The studies involving humans were approved by the Swedish Ethical Review Authority (2019–05498). The studies were conducted in accordance with the local legislation and institutional requirements. The participants provided their written informed consent to participate in this study.

## Author contributions

AB, JL, AG, YE, SE, and IP: study conception and design. AB, JL, AG, AS, DG, SE, and IP: acquisition of data. AB, JL, AG, AS, YE, EH, MR, DG, SE, and IP: analysis and interpretation of data. All authors were involved in the drafting of the manuscript or revising it critically for important intellectual content, and approved the final version to be submitted for publication.

## Funding

This work was supported by the GlaxoSmithKline Investigator-Sponsored Studies (ISS) program, and grants from the Swedish Rheumatism Association (R-969696), King Gustaf V’s 80-year Foundation (FAI-2020-0741), Swedish Society of Medicine (SLS-974449), Nyckelfonden (OLL-974804), Professor Nanna Svartz Foundation (2021-00436), Ulla and Roland Gustafsson Foundation (2021-26), Region Stockholm (FoUI-955483), and Karolinska Institutet. The funders were not involved in the study design, analysis, interpretation of data, the writing of this article, or the decision to submit it for publication.

## Conflict of interest

IP has received research funding and honoraria from Amgen, AstraZeneca, Aurinia Pharmaceuticals, Elli Lilly and Company, Gilead Sciences, GlaxoSmithKline, Janssen Pharmaceuticals, Novartis and F. Hoffmann-La Roche AG.

The remaining authors declare that the research was conducted in the absence of any commercial or financial relationships that could be construed as a potential conflict of interest.

## Publisher’s note

All claims expressed in this article are solely those of the authors and do not necessarily represent those of their affiliated organizations, or those of the publisher, the editors and the reviewers. Any product that may be evaluated in this article, or claim that may be made by its manufacturer, is not guaranteed or endorsed by the publisher.
