# Supplementary material for: Obesity is associated with pain and impaired mobility despite therapy in systemic lupus erythematosus
Source: Front Med (Lausanne). 2023 Aug 24;10:1247354. doi: 10.3389/fmed.2023.1247354 (PMC10484101; doi:10.3389/fmed.2023.1247354)
Supplement: Supplementary file 1 [file Data_Sheet_1.PDF]

Supplementary Table S1. Associations between BMI and HRQoL impairments at baseline, stratified by EQ-5D dimensions.

EQ-5D mobility						
	Estimate	S.E.	P value	OR	95% CI	
					lower	upper
BMI	0.052	0.010	**<0.001**	1.053	1.034	1.073
Black/African American	0.256	0.187	0.170	1.292	0.896	1.866
Asian	-0.404	0.146	**0.006**	0.667	0.500	0.888
Indigenous American	-0.278	0.132	**0.035**	0.757	0.584	0.979
Age	0.014	0.005	**0.004**	1.014	1.004	1.023
SLEDAI-2K	0.056	0.014	**<0.001**	1.057	1.029	1.086
Prednisone eq. dose	0.000	0.006	0.947	1.000	0.988	1.012
**EQ-5D self-care**						
	Estimate	S.E.	P value	OR	95% CI	
					lower	upper
BMI	0.028	0.011	**0.010**	1.029	1.007	1.051
Black/African American	0.317	0.212	0.134	1.374	0.899	2.067
Asian	-0.459	0.200	**0.022**	0.632	0.423	0.929
Indigenous American	-0.142	0.164	0.387	0.867	0.626	1.193
Age	0.008	0.006	0.158	1.008	0.997	1.020
SLEDAI-2K	0.075	0.016	**<0.001**	1.078	1.045	1.113
Prednisone eq. dose	-0.006	0.008	0.458	0.994	0.979	1.009
**EQ-5D usual activities**						
	Estimate	S.E.	P value	OR	95% CI	
					lower	upper
BMI	0.040	0.010	**<0.001**	1.040	1.020	1.061
Black/African American	0.103	0.197	0.602	1.108	0.757	1.641
Asian	-0.782	0.142	**<0.001**	0.457	0.346	0.603
Indigenous American	-0.611	0.131	**<0.001**	0.543	0.419	0.702
Age	0.012	0.005	**0.016**	1.012	1.002	1.022
SLEDAI-2K	0.073	0.014	**<0.001**	1.075	1.046	1.106
Prednisone eq. dose	-0.002	0.006	0.774	0.998	0.986	1.010
**EQ-5D pain/discomfort**						
	Estimate	S.E.	P value	OR	95% CI	
					lower	upper
BMI	0.032	0.013	**0.019**	1.032	1.006	1.060
Black/African American	0.731	0.333	**0.028**	2.078	1.128	4.210
Asian	-0.801	0.163	**<0.001**	0.449	0.326	0.617
Indigenous American	-0.645	0.160	**<0.001**	0.525	0.384	0.719
Age	0.024	0.006	**<0.001**	1.024	1.012	1.037
SLEDAI-2K	0.058	0.018	**0.001**	1.059	1.023	1.098
Prednisone eq. dose	-0.003	0.008	0.673	0.997	0.982	1.012
**EQ-5D anxiety/depression**						
	Estimate	S.E.	P value	OR	95% CI	
					lower	upper
BMI	0.020	0.009	**0.035**	1.020	1.001	1.039
Black/African American	-0.378	0.183	**0.039**	0.685	0.478	0.982
Asian	-0.204	0.138	0.140	0.815	0.621	1.069

Indigenous American	-0.522	0.129	**<0.001**	0.594	0.461	0.763
Age	0.003	0.005	0.558	1.003	0.994	1.012
SLEDAI-2K	0.034	0.013	**0.012**	1.034	1.007	1.062
Prednisone eq. dose	0.015	0.006	**0.015**	1.015	1.003	1.027

Results from logistic regression models. Reference ancestry was White/Caucasian. Statistically significant P values are in bold. BMI: body mass index; CI: confidence interval; eq.: equivalent; HRQoL: health-related quality of life; OR: odds ratio; S.E.; standard error; SLEDAI-2K: Systemic Lupus Erythematosus Disease Activity Index 2000.
