# Supplementary material for: Obesity is associated with pain and impaired mobility despite therapy in systemic lupus erythematosus
Source: Front Med (Lausanne). 2023 Aug 24;10:1247354. doi: 10.3389/fmed.2023.1247354 (PMC10484101; doi:10.3389/fmed.2023.1247354)
Supplement: Supplementary file 2 [file Data_Sheet_2.PDF]

Supplementary Table S2. Associations between BMI categories and HRQoL impairments at baseline, stratified by EQ-5D dimensions.

**EQ-5D mobility**						
	Estimate	S.E.	P value	OR	95% CI	
					lower	upper
Obesity	0.739	0.146	**<0.001**	2.094	1.574	2.792
Pre-obesity	0.352	0.125	**0.005**	1.422	1.113	1.816
Underweight	0.041	0.258	0.874	1.042	0.620	1.714
Black/African American	0.270	0.186	0.148	1.310	0.909	1.891
Asian	-0.440	0.146	**0.003**	0.644	0.483	0.856
Indigenous American	-0.292	0.132	**0.027**	0.747	0.576	0.966
Age	0.014	0.005	**0.003**	1.015	1.005	1.024
SLEDAI-2K	0.055	0.014	**<0.001**	1.057	1.028	1.086
Prednisone eq. dose	-0.001	0.006	0.935	0.999	0.988	1.012
**EQ-5D self-care**						
	Estimate	S.E.	P value	OR	95% CI	
					lower	upper
Obesity	0.486	0.174	**0.005**	1.625	1.152	2.284
Pre-obesity	0.262	0.159	0.099	1.299	0.950	1.770
Underweight	0.535	0.300	0.074	1.708	0.925	3.014
Black/African American	0.310	0.212	0.145	1.363	0.891	2.053
Asian	-0.496	0.200	**0.013**	0.609	0.407	0.894
Indigenous American	-0.140	0.165	0.393	0.869	0.627	1.196
Age	0.009	0.006	0.117	1.009	0.998	1.021
SLEDAI-2K	0.074	0.016	**<0.001**	1.077	1.043	1.111
Prednisone eq. dose	-0.006	0.008	0.475	0.994	0.979	1.009
**EQ-5D usual activities**						
	Estimate	S.E.	P value	OR	95% CI	
					lower	upper
Obesity	0.711	0.157	**<0.001**	2.037	1.502	2.779
Pre-obesity	0.228	0.126	0.070	1.256	0.981	1.608
Underweight	0.059	0.248	0.811	1.061	0.652	1.727
Black/African American	0.109	0.198	0.582	1.115	0.761	1.652
Asian	-0.795	0.141	**<0.001**	0.452	0.342	0.595
Indigenous American	-0.614	0.132	**<0.001**	0.541	0.418	0.701
Age	0.012	0.005	**0.015**	1.012	1.002	1.022
SLEDAI-2K	0.073	0.014	**<0.001**	1.075	1.046	1.106
Prednisone eq. dose	-0.001	0.006	0.810	0.999	0.987	1.011
**EQ-5D pain/discomfort**						
	Estimate	S.E.	P value	OR	95% CI	
					lower	upper
Obesity	0.623	0.219	**0.004**	1.865	1.228	2.911
Pre-obesity	0.261	0.158	0.099	1.298	0.955	1.778
Underweight	0.403	0.301	0.181	1.496	0.847	2.781
Black/African American	0.727	0.333	**0.029**	2.070	1.123	4.195
Asian	-0.817	0.162	**<0.001**	0.442	0.322	0.607
Indigenous American	-0.638	0.160	**<0.001**	0.528	0.386	0.724
Age	0.025	0.006	**<0.001**	1.025	1.012	1.038
SLEDAI-2K	0.057	0.018	**0.002**	1.059	1.022	1.097
Prednisone eq. dose	-0.003	0.008	0.712	0.997	0.983	1.012

EQ-5D anxiety/depression						
	Estimate	S.E.	P value	OR	95% CI	
					lower	upper
Obesity	0.295	0.146	**0.043**	1.343	1.010	1.790
Pre-obesity	0.086	0.123	0.485	1.090	0.856	1.387
Underweight	-0.202	0.242	0.404	0.817	0.506	1.314
Black/African American	-0.369	0.183	**0.044**	0.692	0.483	0.991
Asian	-0.208	0.138	0.131	0.812	0.620	1.064
Indigenous American	-0.527	0.129	**<0.001**	0.590	0.458	0.759
Age	0.003	0.005	0.572	1.003	0.993	1.012
SLEDAI-2K	0.034	0.013	**0.012**	1.034	1.008	1.062
Prednisone eq. dose	0.015	0.006	**0.016**	1.015	1.003	1.027

Results from logistic regression models. Reference ancestry was White/Caucasian. Reference BMI category was normal weight. Statistically significant P values are in bold. BMI: body mass index; CI: confidence interval: eq.: equivalent; HRQoL: health-related quality of life; OR: odds ratio; S.E.; standard error; SLEDAI-2K: Systemic Lupus Erythematosus Disease Activity Index 2000.
