# Supplementary material for: Obesity is associated with pain and impaired mobility despite therapy in systemic lupus erythematosus
Source: Front Med (Lausanne). 2023 Aug 24;10:1247354. doi: 10.3389/fmed.2023.1247354 (PMC10484101; doi:10.3389/fmed.2023.1247354)
Supplement: Supplementary file 3 [file Data_Sheet_3.PDF]

Supplementary Table S3. Associations between BMI and HRQoL impairments at week 52, stratified by EQ-5D dimensions.

**EQ-5D mobility**						
	Estimate	S.E.	P value	OR	95% CI	
					lower	upper
Mean BMI week 0–52	0.028	0.011	**0.009**	1.028	1.007	1.050
BL EQ-5D MOB problems	1.883	0.123	**<0.001**	6.576	5.177	8.391
Black/African American	-0.070	0.213	0.742	0.932	0.613	1.412
Asian	-0.205	0.175	0.240	0.814	0.577	1.145
Indigenous American	-0.219	0.156	0.161	0.803	0.590	1.090
Age	0.034	0.006	**<0.001**	1.035	1.023	1.046
Mean SLEDAI-2K week 0–52	0.106	0.017	**<0.001**	1.112	1.075	1.151
Prednisone eq. dose at baseline	0.011	0.007	0.120	1.011	0.997	1.025
Belimumab use	-0.269	0.127	**0.034**	0.764	0.596	0.981
**EQ-5D self-care**						
	Estimate	S.E.	P value	OR	95% CI	
					lower	upper
Mean BMI week 0–52	0.026	0.013	**0.048**	1.026	1.000	1.053
BL EQ-5D SC problems	2.516	0.158	**<0.001**	12.383	9.108	16.937
Black/African American	0.004	0.270	0.988	1.004	0.585	1.686
Asian	-0.105	0.235	0.654	0.900	0.563	1.416
Indigenous American	-0.011	0.200	0.955	0.989	0.666	1.457
Age	0.021	0.007	**0.004**	1.021	1.007	1.036
Mean SLEDAI-2K week 0–52	0.099	0.020	**<0.001**	1.104	1.061	1.149
Prednisone eq. dose at baseline	-0.001	0.009	0.943	0.999	0.981	1.018
Belimumab use	-0.344	0.162	**0.034**	0.709	0.517	0.975
**EQ-5D usual activities**						
	Estimate	S.E.	P value	OR	95% CI	
					lower	upper
Mean BMI week 0–52	0.018	0.010	0.083	1.018	0.998	1.038
BL EQ-5D UA problems	1.698	0.118	**<0.001**	5.464	4.341	6.907
Black/African American	0.080	0.202	0.692	1.083	0.730	1.615
Asian	-0.470	0.160	**0.003**	0.625	0.456	0.855
Indigenous American	-0.449	0.145	**0.002**	0.638	0.480	0.847
Age	0.027	0.005	**<0.001**	1.028	1.017	1.038
Mean SLEDAI-2K week 0–52	0.081	0.017	**<0.001**	1.084	1.050	1.120
Prednisone eq. dose at baseline	0.003	0.007	0.699	1.003	0.990	1.016
Belimumab use	-0.055	0.119	0.646	0.947	0.749	1.197
**EQ-5D pain/discomfort**						
	Estimate	S.E.	P value	OR	95% CI	
					lower	upper
Mean BMI week 0–52	0.032	0.012	**0.005**	1.033	1.010	1.057
BL EQ-5D PD problems	1.683	0.139	**<0.001**	5.382	4.105	7.087
Black/African American	0.142	0.234	0.545	1.152	0.736	1.849
Asian	-0.311	0.156	**0.047**	0.733	0.540	0.996
Indigenous American	-0.129	0.150	0.390	0.879	0.656	1.181
Age	0.027	0.006	**<0.001**	1.028	1.016	1.039
Mean SLEDAI-2K week 0–52	0.089	0.018	**<0.001**	1.093	1.055	1.134
Prednisone eq. dose at baseline	-0.014	0.007	**0.047**	0.987	0.974	1.000

Belimumab use	-0.283	0.128	**0.027**	0.754	0.586	0.966
**EQ-5D anxiety/depression**						
	Estimate	S.E.	P value	OR	95% CI	
					lower	upper
Mean BMI week 0–52	0.008	0.010	0.463	1.008	0.988	1.028
BL EQ-5D AD problems	2.039	0.116	**<0.001**	7.686	6.131	9.681
Black/African American	-0.171	0.210	0.415	0.843	0.558	1.271
Asian	-0.058	0.156	0.709	0.944	0.695	1.280
Indigenous American	0.006	0.145	0.965	1.006	0.757	1.338
Age	0.017	0.005	**0.001**	1.017	1.007	1.028
Mean SLEDAI-2K week 0–52	0.076	0.016	**<0.001**	1.079	1.045	1.115
Prednisone eq. dose at baseline	0.000	0.007	0.955	1.000	0.987	1.013
Belimumab use	-0.218	0.119	**0.068**	0.804	0.636	1.016

Results from logistic regression models. Reference ancestry was White/Caucasian. Statistically significant P values are in bold. AD: anxiety/depression; BL: baseline; BMI: body mass index; CI: confidence interval; eq.: equivalent; HRQoL: health-related quality of life; MOB: mobility; OR: odds ratio; PD: pain/discomfort; SC: self-care; S.E.; standard error; SLEDAI-2K: Systemic Lupus Erythematosus Disease Activity Index 2000; UA: usual activities.
