# Supplementary material for: Obesity is associated with pain and impaired mobility despite therapy in systemic lupus erythematosus
Source: Front Med (Lausanne). 2023 Aug 24;10:1247354. doi: 10.3389/fmed.2023.1247354 (PMC10484101; doi:10.3389/fmed.2023.1247354)
Supplement: Supplementary file 4 [file Data_Sheet_4.PDF]

Supplementary Table S4. Associations between BMI categories and HRQoL impairments at week 52, stratified by EQ-5D dimensions.

**EQ-5D mobility**						
	Estimate	S.E.	P value	OR	95% CI	
					lower	upper
Obesity based on mean BMI week 0–52	0.353	0.164	**0.031**	1.423	1.032	1.960
Pre-obesity based on mean BMI week 0–52	0.386	0.147	**0.009**	1.471	1.102	1.962
Underweight based on mean BMI week 0–52	-0.086	0.333	0.796	0.917	0.464	1.727
BL EQ-5D MOB problems	1.897	0.123	**<0.001**	6.668	5.249	8.511
Black/African American	-0.078	0.213	0.714	0.925	0.608	1.401
Asian	-0.222	0.175	0.203	0.801	0.567	1.125
Indigenous American	-0.245	0.157	0.118	0.783	0.575	1.063
Age	0.034	0.006	**<0.001**	1.034	1.023	1.046
Mean SLEDAI-2K week 0–52	0.107	0.017	**<0.001**	1.113	1.076	1.152
Prednisone eq. dose at baseline	0.010	0.007	0.146	1.010	0.996	1.025
Belimumab use	-0.271	0.127	**0.033**	0.762	0.594	0.978
**EQ-5D self-care**						
	Estimate	S.E.	P value	OR	95% CI	
					lower	upper
Obesity based on mean BMI week 0–52	0.236	0.211	0.263	1.266	0.835	1.912
Pre-obesity based on mean BMI week 0–52	0.416	0.188	**0.027**	1.515	1.047	2.189
Underweight based on mean BMI week 0–52	0.512	0.389	0.187	1.669	0.750	3.466
BL EQ-5D SC problems	2.533	0.159	**<0.001**	12.588	9.249	17.238
Black/African American	-0.007	0.270	0.980	0.993	0.579	1.668
Asian	-0.180	0.235	0.443	0.835	0.523	1.314
Indigenous American	-0.041	0.199	0.835	0.959	0.646	1.413
Age	0.023	0.007	**0.002**	1.023	1.008	1.037
Mean SLEDAI-2K week 0–52	0.099	0.020	**<0.001**	1.104	1.061	1.149
Prednisone eq. dose at baseline	-0.001	0.009	0.933	0.999	0.981	1.017
Belimumab use	-0.343	0.162	**0.034**	0.709	0.517	0.975
**EQ-5D usual activities**						
	Estimate	S.E.	P value	OR	95% CI	
					lower	upper
Obesity based on mean BMI week 0–52	0.167	0.156	0.284	1.182	0.871	1.605
Pre-obesity based on mean BMI week 0–52	0.195	0.139	0.158	1.216	0.926	1.595
Underweight based on mean BMI week 0–52	0.208	0.287	0.468	1.232	0.695	2.148
BL EQ-5D UA problems	1.710	0.119	**<0.001**	5.526	4.389	6.990
Black/African American	0.084	0.202	0.677	1.088	0.733	1.622
Asian	-0.502	0.160	**0.002**	0.605	0.441	0.827
Indigenous American	-0.465	0.145	**0.001**	0.628	0.472	0.833
Age	0.028	0.005	**<0.001**	1.028	1.018	1.039
Mean SLEDAI-2K week 0–52	0.081	0.017	**<0.001**	1.084	1.050	1.121
Prednisone eq. dose at baseline	0.002	0.007	0.719	1.002	0.989	1.016
Belimumab use	-0.056	0.119	0.638	0.945	0.748	1.195
**EQ-5D pain/discomfort**						
	Estimate	S.E.	P value	OR	95% CI	
					lower	upper
Obesity based on mean BMI week 0–52	0.428	0.181	**0.018**	1.534	1.080	2.201
Pre-obesity based on mean BMI week 0–52	-0.015	0.143	0.914	0.985	0.745	1.303

Underweight based on mean BMI week 0–52	-0.089	0.278	0.749	0.915	0.533	1.593
BL EQ-5D PD problems	1.685	0.139	**<0.001**	5.391	4.110	7.102
Black/African American	0.173	0.234	0.461	1.188	0.760	1.905
Asian	-0.335	0.156	**0.032**	0.715	0.527	0.972
Indigenous American	-0.123	0.150	0.412	0.884	0.660	1.189
Age	0.029	0.006	**<0.001**	1.029	1.018	1.041
Mean SLEDAI-2K week 0–52	0.089	0.018	**<0.001**	1.093	1.055	1.134
Prednisone eq. dose at baseline	-0.013	0.007	**0.049**	0.987	0.974	1.000
Belimumab use	-0.279	0.128	**0.029**	0.756	0.588	0.970
**EQ-5D anxiety/depression**						
	Estimate	S.E.	P value	OR	95% CI	
					lower	upper
Obesity based on mean BMI week 0–52	0.114	0.159	0.471	1.121	0.821	1.532
Pre-obesity based on mean BMI week 0–52	0.041	0.139	0.767	1.042	0.794	1.368
Underweight based on mean BMI week 0–52	0.374	0.285	0.189	1.454	0.829	2.539
BL EQ-5D AD problems	2.048	0.117	**<0.001**	7.752	6.182	9.769
Black/African American	-0.173	0.210	0.411	0.841	0.557	1.270
Asian	-0.081	0.155	0.601	0.922	0.680	1.250
Indigenous American	0.003	0.145	0.982	1.003	0.755	1.335
Age	0.018	0.005	**0.001**	1.018	1.007	1.029
Mean SLEDAI-2K week 0–52	0.076	0.016	**<0.001**	1.079	1.045	1.115
Prednisone eq. dose at baseline	0.000	0.007	0.975	1.000	0.987	1.013
Belimumab use	-0.220	0.119	0.065	0.802	0.634	1.014

Results from logistic regression models. Reference ancestry was White/Caucasian. Reference BMI category was normal weight. Statistically significant P values are in bold. AD: anxiety/depression; BL: baseline; BMI: body mass index; CI: confidence interval; eq.: equivalent; HRQoL: health-related quality of life; MOB: mobility; OR: odds ratio; PD: pain/discomfort; SC: self-care; S.E.; standard error; SLEDAI-2K: Systemic Lupus Erythematosus Disease Activity Index 2000; UA: usual activities.
